# When the second comes first– rhabdomyosarcoma preceding heritable retinoblastoma– a case report

**DOI:** 10.1186/s12886-024-03307-x

**Published:** 2024-01-30

**Authors:** Devjyoti Tripathy, Alexandre Moulin, Jacques Bijon, Carole Gengler, Maja Beck-Popovic, Francis L. Munier, Christina Stathopoulos

**Affiliations:** 1 LV Prasad Eye Institute, MTC Campus, Bhubaneswar, Odisha India; 2 Fondation Asile des Aveugles, Jules-Gonin Eye Hospital, University of Lausanne, Lausanne, Switzerland; 3 Unit of Pathology, Centre Hospitalier Universitaire Vaudois, Lausanne, Switzerland; 4 Unit of Pediatric Hematology-Oncology, Centre Hospitalier Universitaire Vaudois, Lausanne, Switzerland

**Keywords:** Retinoblastoma, Rhabdomyosarcoma, Non-ocular, Second primary tumors, Case report

## Abstract

**Background:**

Retinoblastoma (rb) is the most frequent intraocular tumor, accounting for 3% of all childhood cancers. Heritable rb survivors are germline carriers for an RB1 mutation and have a lifelong risk to develop non-ocular second primary tumors (SPTs) involving multiple other organs like the bones, soft tissues, or skin. These SPTs usually become manifest several years succeeding the diagnosis of rb. In our instance, however, a non-ocular SPT presented prior to the diagnosis of heritable rb.

**Case presentation:**

We report a rare case of a monozygotic twin who presented with primary rhabdomyosarcoma (RMS) preceding the manifestation of heritable rb. The rb was diagnosed when the child developed strabismus while already on therapy for the RMS. The child underwent therapy for both as per defined treatment protocols. The rb regressed well on treatment, but the RMS relapsed and the child developed multiple refractory metastatic foci and succumbed to his disease.

**Conclusions:**

Non-ocular SPTs like sarcomas are usually known to manifest in heritable rb survivors with a lag of two to three decades (earlier if exposure to radiation is present) from the presentation of the rb. However, in our case, this seemed to be reversed with the RMS being manifest at an unusual early age and the rb being diagnosed at a later point in time.

## Background

Retinoblastoma (rb) is the most frequent intraocular tumor, accounting for 3% of all childhood cancers. Retinoblastoma survivors with a germline *RB1* mutation are at lifetime risk to develop various types of non-ocular second primary tumors (SPTs) in diverse anatomic locations, such as the bones, the soft tissue or the skin [1]. This risk is further increased after external beam radiotherapy and, to a lesser extent, chemotherapy [2]. Herein we report a rare case of a monozygotic twin presenting with primary rhabdomyosarcoma preceding the diagnosis of heritable rb.

## Case presentation

**Fig. 1:**
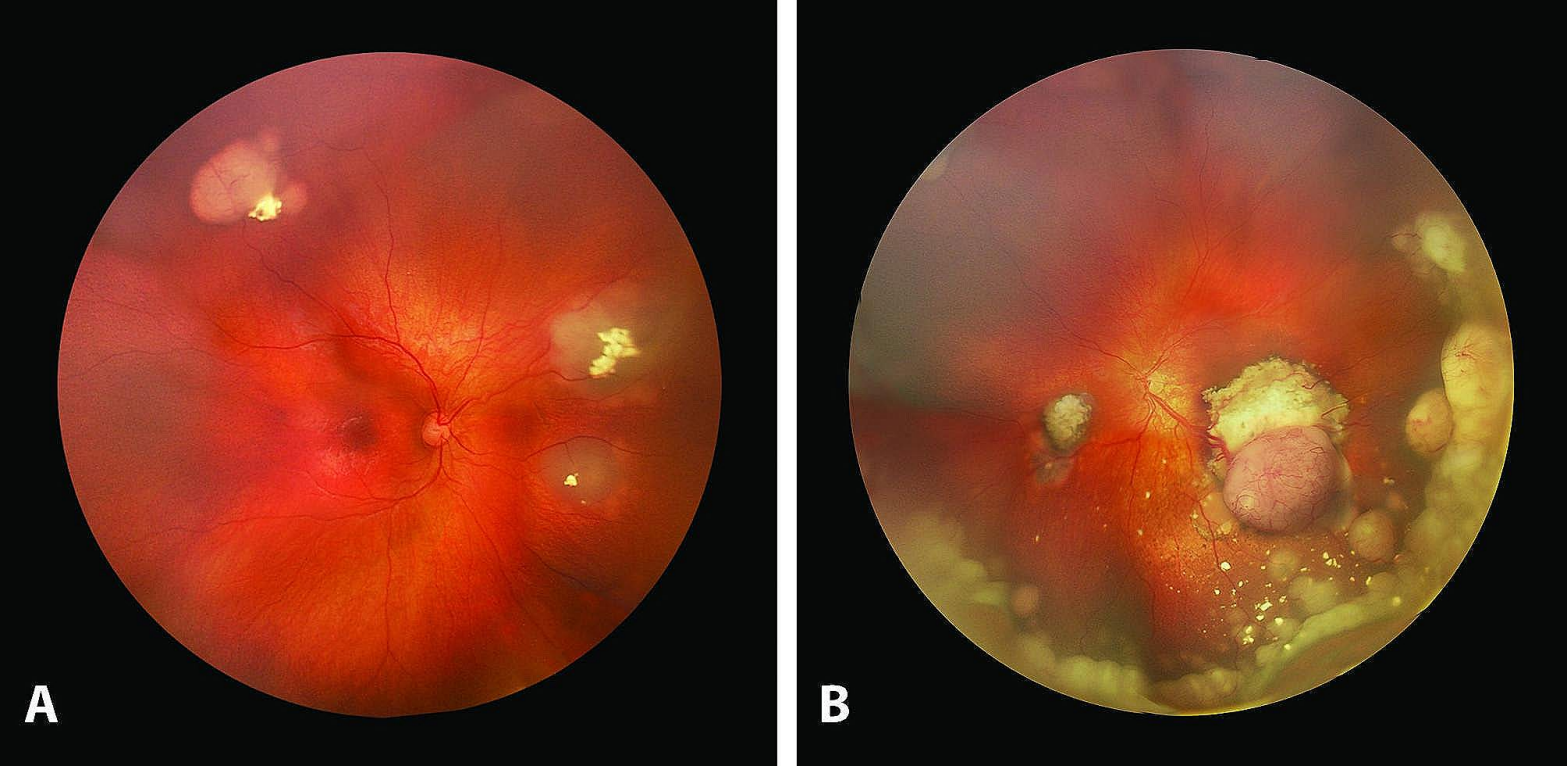
Fundus pictures of a 14-month-old boy developing strabismus of the left eye while under systemic chemotherapy for an alveolar RMS with the right eye showing three partially regressed endophytic tumors with focal intralesional calcifications **(A)** and the left eye showing two relapsing tumors with diffuse subretinal seeding reaching the inferior ora serrata **(B)**

**Fig. 2:**
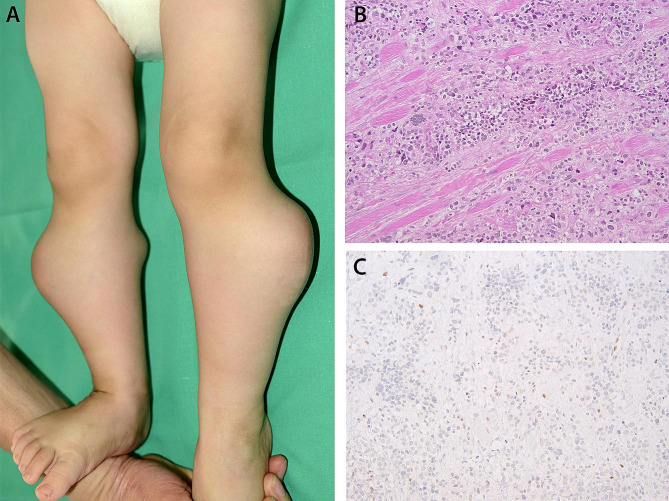
Bilateral nodular multifocal deformations of both lower extremities from alveolar RMS metastases **(A)** confirmed on histopathology showing nests and sheets of undifferentiated round tumor cells with rhabdoid features infiltrating the striated muscle (hematoxylin and eosin, magnification X126) **(B)**. Immunostaining of the tumor tissue displaying *RB1* loss in most of the tumor nuclei (magnification X126) **(C)**

A 14-month-old boy developed left eye strabismus following sustentacular systemic vinblastine, cyclophosphamide, and local radiotherapy for an alveolar RMS (*PAX3-FOXO1* positive) of the right buttock diagnosed elsewhere at the age of 5 months. Previous treatments included systemic polychemotherapy (carboplatin, doxorubicin, vincristine, ifosfamide, etoposide and actinomycin) (CWS-2009 protocol), five cycles given prior, and four after incomplete surgical resection of the tumor. Ophthalmic evaluation revealed bilateral rb, that was also present in the younger asymptomatic monozygotic fellow twin. Fundus examination of the 3-year older brother and the parents was normal. Both twins were referred to Lausanne for further treatment. Examination under anesthesia of the elder one revealed three partially regressed tumors in the right eye (OD) and two relapsing tumors in the left eye (OS) associated with diffuse subretinal seeding, consistent with International Intraocular Retinoblastoma Classification (IIRC) Group B in OD and group D in OS (Fig. 1). Intraocular tumor regression was achieved with one intra-arterial melphalan (3.7 mg) in OS and bilateral focal treatments (5 sessions of cryotherapy and/or thermotherapy given over 5 months). However, about a year following the diagnosis of rb, the child unfortunately developed refractory multifocal metastatic RMS involving both lower extremities (Fig. 2A), the lungs, and the brain confirmed on histopathology and immunostaining (Fig. 2B, C), and succumbed to his disease. The fellow twin had rb IIRC group B in OD and group D in OS treated with one cycle of systemic chemotherapy followed by three intra-arterial melphalan in OS together with bilateral focal treatments and remained tumor-free at 6 years follow-up.

Both twins were found to be carriers of a *RB1* loss of function mutation (NM_000321.2:c.1128-1G > A:p.(Thr377 Leufs*3). The father was a 10% mosaic healthy carrier.

## Discussion and conclusions

Most rb patients are diagnosed before the age of 3 years, those with a known family history of rb and bilateral rb being diagnosed usually earlier than the unilateral form (4 months and 12 months respectively versus 24 months) [3]. Germline carriers for a *RB1* mutation have a 3.2% risk to develop a midline intracranial tumor (MIT) (most commonly a pinealoblastoma) referred to as trilateral rb and a lifelong risk to develop SPTs which cumulative incidence is reported to be 3.7% at the age of 10 years and 17.7% at the age of 35 years [4, 5]. The most prevalent SPTs are sarcoma (68%) followed by carcinoma (14%) and melanoma (8%) [1]. In contrast to MIT that can occur before or at time of the rb diagnosis-, other SPT cases follow the rb diagnosis, with a latency ranging 1–55 years [1]. 

Rhabdomyosarcoma is the most common soft tissue sarcoma in children, accounting for about 3% of childhood cancers [6]. In rb survivors, rhabdomyosarcoma is the second most frequent sarcoma after osteosarcoma with over 85% of cases occurring in the head and neck within the field of irradiation [1]. Alveolar RMS is a subtype of RMS with an incidence of 1 per million children and adolescents. Most cases occur sporadically with no genetic predisposition and are associated with a balanced chromosomal translocation t(2;13)(q35;q14) or t(1:13)(p36;q14) generating the PAX3 or PAX7-FOXO1 fusion variant exhibiting oncogenic activity [6]. Alveolar RMS most commonly presents in the pediatric and adolescent age group (typically between 10 and 20 years), which is at a later age compared to its commoner counterpart embryonal RMS (presenting typically between birth and 5 years), and is known to have a predilection for the extremities and the perineal region [7]. 

Herein we report a case of bilateral rb, partially regressed during systemic chemotherapy given for an alveolar RMS, which only became symptomatic on relapse once the systemic polychemotherapy was stopped. Though of extremely rare occurrence, this case illustrates that a non-ocular SPT may manifest at an unusually early age in a patient with heritable rb and may precede the diagnosis of the rb itself. The awareness of this possibility may be of help to onco-pediatricians involved with the management of such cases.

## Data Availability

Available with the corresponding author.

## Abbreviations

rb/RB: retinoblastoma
SPT: second primary tumor
RMS: rhabdomyosarcoma
MIT: midline intracranial tumor
CWS: Cooperative Weichteilsarkom Studiengruppe
IIRC: International Intraocular Retinoblastoma Classification
OD: oculus dexter
OS: oculus sinister

## References

[1] Woo KI, Harbour JW (2010). Review of 676 second primary tumors in patients with retinoblastoma: association between age at onset and tumor type. Arch Ophthalmol.

[2] Temming P, Arendt M, Viehmann A (2017). Incidence of second cancers after radiotherapy and systemic chemotherapy in heritable retinoblastoma survivors: a report from the German reference center. Pediatr Blood Cancer.

[3] Dimaras H, Corson TW, Cobrinik D (2015). Retinoblastoma. Nat Rev Dis Primers.

[4] de Jong MC, Kors WA, de Graaf P (2014). Trilateral retinoblastoma: a systematic review and meta-analysis. Lancet Oncol.

[5] Moll AC, Imhof SM, Bouter LM, et al. Second primary tumors in patients with hereditary retinoblastoma: a register-based follow-up study, 1945–1994. Int J Cancer. 1996;67(4):515–9. 10.1002/(SICI)1097-0215(19960807)67:4%3C515::AID-IJC9%3E3.0.CO;2-V. 10.1002/(SICI)1097-0215(19960807)67:4<515::AID-IJC9>3.0.CO;2-V 8759610

[6] Skapek SX, Ferrari A, Gupta AA (2019). Rhabdomyosarcoma. Nat Rev Dis Primers.

[7] Agaram NP (2022). Evolving classification of rhabdomyosarcoma. Histopathology.

